# Chronic obstructive pulmonary disease: a complex comorbidity of lung cancer

**DOI:** 10.15256/joc.2011.1.5

**Published:** 2011-12-27

**Authors:** Derek Grose, Robert Milroy

**Affiliations:** ^1^Beatson West of Scotland Cancer Centre, Glasgow, Scotland, UK; ^2^Glasgow Royal Infirmary, Glasgow, Scotland, UK

**Keywords:** chronic obstructive pulmonary disease (COPD), comorbidity, inflammation, lung cancer, oxidative stress, pulmonary

## Abstract

Chronic obstructive pulmonary disease (COPD) is a major burden throughout the world. It is associated with a significantly increased incidence of lung cancer and may influence treatment options and outcome. Impaired lung function confirming COPD is an independent risk factor for lung cancer. Oxidative stress and inflammation may be a key link between COPD and lung cancer, with numerous molecular markers being analysed to attempt to understand the pathway of lung cancer development. COPD negatively influences the ability to deliver radical treatment options, so attempts must be made to look for alternative methods of treating lung cancer, while aiming to manage the underlying COPD. Detailed assessment and management plans utilising the multidisciplinary team must be made for all lung cancer patients with COPD to provide the best care possible.

Journal of Comorbidity 2011;1:45–50

## Introduction

Chronic obstructive pulmonary disease (COPD) is characterized, according to the Global Initiative for Chronic Obstructive Lung Disease^®^ (GOLD), as “chronic airflow limitation and a range of pathological changes in the lung, some significant extrapulmonary effects, and important comorbidities which may contribute to the severity of the disease in individual patients” [1, 2]. The airflow limitation characterizing COPD is not fully reversible, is usually progressive, and results from an abnormal inflammatory response to noxious particles or gases in the lungs [2, 3].

A preventable and treatable disease, COPD is a costly burden to healthcare systems as well as an important cause of morbidity and mortality [3]. Worldwide, approximately 10% of adults have COPD graded as moderate severity or worse [forced expiratory volume in 1 second (FEV_1_) <80%] [4], and COPD is an increasing problem in the developing world [5]. Although the risk of COPD is increased by exposure to air pollution, occupational hazards, and infections, the single most important risk factor is cigarette smoking [3]. It therefore comes as no surprise that COPD is a commonly encountered comorbidity in patients with lung cancer [6–8]. Indeed, recent studies have shown that COPD affects 50–90% of lung cancer patients [8, 9]. Moreover, patients with COPD are three to four times more likely to develop lung cancer compared with smokers with normal lung function [10, 11], and lung cancer is a major cause of mortality in COPD patients, particularly in those with mild or moderate disease [12]. However, it must be noted, that at least some of the association may be related to ‘detection bias’ in that subclinical COPD may be diagnosed during pre-assessment for lung surgery or radiotherapy in a lung cancer patient.

COPD, in addition to many other comorbidities, has a significant impact upon the ability to deliver recommended treatment and consequently on outcome [13–17]. This is not only the case in radical treatment delivery aiming for cure but also in (the far more common) situations where palliative chemotherapy and/or radiotherapy are being considered to improve both duration and quality of life.

COPD has long been recognized as an indicator of a high risk of complications after lung resection [18, 19]. For example, in patients with lung cancer and COPD who undergo surgery, postoperative pneumonia and tracheostomy are more frequent in patients with COPD than in those without [20]. Moreover, the presence of COPD significantly increases the risk of cardiac dysrhythmias, specifically supraventricular tachycardia [21]. Mortality rates are significantly higher in lung cancer patients who have postoperative pulmonary complications than in those who do not [18], and in comparison with lung cancer patients who do not have COPD, those with COPD have poorer long-term survival as a result of respiratory insufficiency [22], a higher rate of recurrence of the lung cancer [20], and poorer survival after surgery [23]. The clear link between the severity of the COPD and survival confirms COPD as a key prognostic factor in patients with lung cancer [23, 24].

## Pathophysiology

**Figure 1:**
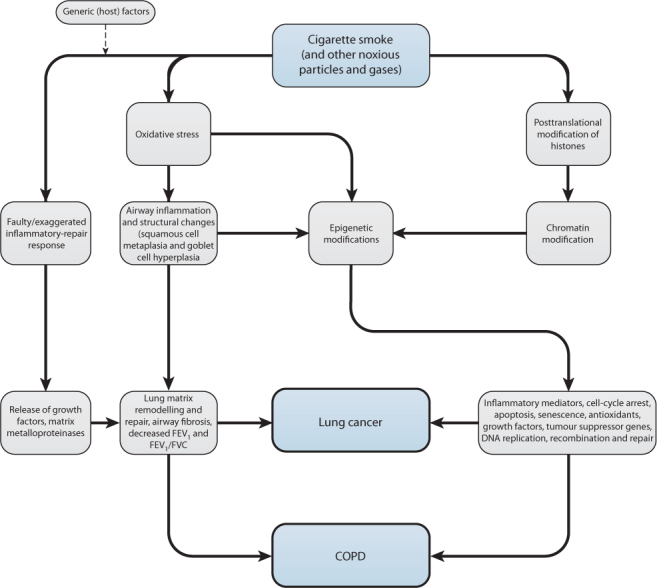
Pathophysiology of chronic obstructive pulmonary disease (COPD) and lung cancer. FEV_1_, forced expiratory volume in 1 second; FVC, forced vital capacity.

Impaired lung function, as indicated by a reduced baseline FEV_1_ and reduced FEV_1_ to forced vital capacity (FVC) ratio – that is, COPD – has been shown in several studies to be an independent risk factor for lung cancer [e.g. 8, 25, 26] (Figure 1). The risk of lung cancer is at least twice as high [11, 27, 28] and may be up to six times as high [8] in individuals with COPD as in those without COPD. More than 80% of cases of lung cancer and COPD can be attributed to exposure to cigarette smoke, which causes oxidative stress and inflammation in the lung [29, 30]. Oxidative stress and inflammation in turn lead to epigenetic alterations mediated by chromatin-modifying enzymes (histone acetyltransferases, deacetylases, methyltransferases, and demethylases) – which have key roles in functions such as expression of inflammatory mediators, cell-cycle arrest, apoptosis, responses to antioxidants and stress, and replication, recombination, and repair of DNA – and the resulting chromatin remodelling is likely to be at the heart of the link between COPD and lung cancer [29].

At the molecular biological level, there is emerging evidence that COPD and lung cancer are linked by a faulty inflammatory-repair response to cigarette smoke or other airborne pollutants [30]. The increased release of growth factors and matrix metalloproteinases resulting from an exaggerated inflammatory response leads to lung matrix remodelling, including an epithelial to mesenchymal transition – a type of malignant transformation seen in several cancers as well as lung cancer, but also seen in COPD [8, 9, 30, 31]. Factors participating in lung matrix remodelling include inflammatory cytokines such as interleukin 6 [8, 9, 30, 31] and those involved in oxidative stress and ineffective DNA repair [32]. Deregulation of the phosphatidylinositol 3-kinase pathway has been shown to be an early event in the development of lung cancer [33], and altered signalling via the epidermal growth factor receptor may lead to the development of lung cancer in patients with COPD [34].

In efforts to explain why lung cancer develops in only 10–15% of smokers, much recent work has focused on the roles of an aberrant inflammatory response and genetic susceptibility in lung carcinogenesis and COPD [29, 30, 35–39]. Genetic studies have strongly implicated variation in the 15q chromosomal region, where the nicotinic acetylcholine receptor is encoded [40–47], and the 5p region, where genes encode factors with roles in telomerase production, carcinogenesis, and apoptosis [42].

## Management

Recommendations and guidelines developed in accordance with the American College of Physicians (ACP), American College of Chest Physicians (ACCP), American Thoracic Society (ATS) and the European Respiratory Society (ERS) exist for the management of stable COPD [1, 48, 49]. The guidelines proposed by these colleges and societies are summarized in Table 1.

Factors strongly linked to COPD – lung function and performance status – determine whether lung cancer patients with COPD are able to undergo curative surgery or radical radiotherapy [50]. Adequate lung function (evaluated by spirometry) is a prerequisite for potentially curative surgery, and a pre-operative FEV_1_ >1.5 L in patients undergoing a lobectomy or >2.0 L in those undergoing a pneumonectomy is associated with a mortality rate <5% [50]. However, it is essential to evaluate the percentage predicted FEV_1_ as well as the absolute value. In borderline cases, cardiopulmonary exercise testing can be useful in decision making [51]. Although mild COPD does not necessarily preclude definitive treatment of lung cancer, severe COPD may, for example, make the lung cancer inoperable because the patient has low cardiopulmonary reserve [20, 22] and an increased risk of perioperative pulmonary complications [19, 21, 22]. This may, in part, be partially due to severely impaired endothelial repair mechanisms [52]. These observations may explain why lung cancer patients have been more likely to receive non-surgical treatment (i.e. radiotherapy rather than surgery) if they have significant COPD [7, 53]. Indeed, a report from Japan concluded that the main therapeutic goal for lung cancer patients with COPD should be to achieve quality of life improvement through palliative care [54]. However, one study suggests that lobectomy for lung cancer can achieve a better outcome in COPD patients than in non-COPD patients [55]. Furthermore, given the limitations and poor outcomes of non-surgical treatment for COPD, more inclusive surgical criteria have been suggested [56], and alternative surgical techniques (anatomical segmentectomy, lobectomy by video-assisted thoracoscopic surgery) explored [57]. Quality of life after lobectomy has been shown to be similar in COPD and non-COPD patients [58], and recently, use of bronchodilators, such as tiotropium, has been shown to improve surgical outcomes in patients with COPD and lung cancer [34, 59].

One encouraging scenario is the emergence of non-surgical alternatives for radical treatment of localized curable lung cancers in the form of modern radiotherapy techniques, such as stereotactic body radiotherapy (SBRT). A number of studies have been published indicating excellent outcome in medically inoperable, otherwise resectable, lung cancer patients [60, 61]. It is noteworthy that even relatively severe COPD appears to have little effect on the outcome of such patients [62].

It is important to consider that the vast majority of lung cancer patients will present with locally advanced or metastatic disease, making cure very highly unlikely. In these patients, the commonest treatment is for palliative chemotherapy or radiotherapy. There is very little in the literature indicating a direct impact of COPD upon delivery of chemotherapy. However, COPD often negatively impacts upon performance status, which is closely linked to both tolerance and benefit of palliative chemotherapy [63–65].

An important aspect of treatment of COPD and lung cancer concerns possible ‘spillover’ of inflammatory mediators from the lung, which may lead to extrapulmonary effects [66]. The ‘spillage’ can be treated with anti-inflammatory agents (preferably inhaled, to avoid risk of systemic side-effects), to suppress pulmonary inflammation. Examples include corticosteroids, long-acting b_2_ agonists, and theophylline. Inflammation associated with COPD may also be reduced by treatment with statins, angiotensin-converting enzyme inhibitors, or peroxisome proliferator-activated agonists [66]. Statins may be particularly beneficial in patients with COPD because they suppress inflammatory and matrix remodelling pathways, and they target both pulmonary and systemic inflammation [31]. Treatments of the future may target matrix metalloproteinases [67] or the arylhydrocarbon receptor [68], or may consist of cell-based therapies using embryonic or adult stem cells [69].

## Concluding remarks

Both COPD and lung cancer are rising worldwide in incidence and are significant causes of morbidity and mortality, imposing a significant burden on healthcare systems throughout both the developed and developing world. Nearly 40,000 new cases of lung cancer were reported in 2007 in the UK alone [70]. Furthermore, with the increase in smoking and increasing life expectancy in the developing world, lung cancer is likely to only increase as a burden on the health services of developing countries in the future.

It is clear that COPD and lung cancer are closely linked entities with each having a significant detrimental impact upon the other. This ranges from increased incidence of lung cancer in patients with COPD, through to inability to deliver radical therapy and increased complications following surgery.

It is essential that a careful and complete evaluation of all comorbidity, but in particular COPD, should be made in all patients with lung cancer to enable an optimal individualized treatment plan [14, 71] coordinated by the multidisciplinary clinical care team [50].

It is only by addressing this significant challenge of carefully assessing and treating patients with overlapping comorbid conditions that we will be able to develop individualized treatments for patients and improve upon the poor outlook for the majority of our lung cancer patients.

## Figures and Tables

**Table 1:** Guideline recommendations for the management of chronic obstructive pulmonary disease (COPD) [1, 48, 49].

Recommendation		Guideline
1.	Stable COPD patients with respiratory symptoms and FEV_1_ between 60 and 80% predicted may receive treatment with inhaled bronchodilators	ACP, ACCP, ATS, ERS
2.	Symptomatic patients with stable COPD and FEV_1_ <60% predicted may receive treatment with inhaled bronchodilators, monotherapy using either long-acting, inhaled b agonists or long-acting, inhaled anticholinergics or combination inhaled therapies using long-acting b agonists, long-acting anticholinergics, or corticosteroids	ACP, ACCP, ATS, ERS
3.	Pulmonary rehabilitation should be prescribed for symptomatic patients with an FEV_1_ <50% predicted, and considered for symptomatic or exercise-limited patients with an FEV_1_ >50% predicted	ACP, ACCP, ATS, ERS
4.	For COPD patients with severe resting hypoxaemia (PaO_2_ ≤55 mm Hg or SpO_2_ ≤88%), continuous oxygen therapy is recommended	ACP, ACCP, ATS, ERS
5.	The GOLD committee broadly agrees with the above recommendations but advocates the use of inhaled glucocorticosteroids and bronchodilators for symptomatic COPD patients with FEV_1_ <50% predicted and repeated exacerbations	GOLD

ACCP, American College of Chest Physicians; ACP, American College of Physicians; ATS, American Thoracic Society; ERS, European Respiratory Society; FEV_1_, forced expiratory volume in 1 second; GOLD, Global Initiative for Chronic Obstructive Lung Disease^®^; PaO_2_, partial pressure of oxygen in arterial blood; SpO_2,_ saturation of peripheral oxygen.
